# CTPS1 promotes malignant progression of triple-negative breast cancer with transcriptional activation by YBX1

**DOI:** 10.1186/s12967-021-03206-5

**Published:** 2022-01-06

**Authors:** Yuxiang Lin, Jie Zhang, Yan Li, Wenhui Guo, Lili Chen, Minyan Chen, Xiaobin Chen, Wenzhe Zhang, Xuan Jin, Meichen Jiang, Han Xiao, Chuan Wang, Chuangui Song, Fangmeng Fu

**Affiliations:** 1 Department of Breast Surgery, Fujian Medical University Union Hospital, No.29, Xin Quan Road, Gulou District, Fuzhou, 350001 Fujian China; 2 Department of General Surgery, Fujian Medical University Union Hospital, Fuzhou, 350001 Fujian China; 3 Breast Cancer Institute, Fujian Medical University, Fuzhou, Fujian China; 4 Department of Pathology, Fujian Medical University Union Hospital, Fuzhou, 350001 Fujian China

**Keywords:** Triple-negative breast cancer, CTPS1, Progression, YBX1, Transcriptional activation

## Abstract

**Background:**

Cytidine nucleotide triphosphate synthase 1 (CTPS1) is a CTP synthase which play critical roles in DNA synthesis. However, its biological regulation and mechanism in triple-negative breast cancer (TNBC) has not been reported yet.

**Methods:**

The expression of CTPS1 in TNBC tissues was determined by GEO, TCGA databases and immunohistochemistry (IHC). The effect of CTPS1 on TNBC cell proliferation, migration, invasion, apoptosis and tumorigenesis were explored in vivo and in vitro. In addition, the transcription factor Y-box binding protein 1 (YBX1) was identified by bioinformatics methods, dual luciferase reporter and chromatin immunoprecipitation (CHIP) assays. Pearson correlation analysis was utilized to assess the association between YBX1 and CTPS1 expression.

**Results:**

CTPS1 expression was significantly upregulated in TNBC tissues and cell lines. Higher CTPS1 expression was correlated with a poorer disease-free survival (DFS) and overall survival (OS) in TNBC patients. Silencing of CTPS1 dramatically inhibited the proliferation, migration, invasion ability and induced apoptosis of MDA-MB-231 and HCC1937 cells. Xenograft tumor model also indicated that CTPS1 knockdown remarkably reduced tumor growth in mice. Mechanically, YBX1 could bind to the promoter of CTPS1 to promote its transcription. Furthermore, the expression of YBX1 was positively correlated with CTPS1 in TNBC tissues. Rescue experiments confirmed that the enhanced cell proliferation and invasion ability induced by YBX1 overexpression could be reversed by CTPS1 knockdown.

**Conclusion:**

Our data demonstrate that YBX1/CTPS1 axis plays an important role in the progression of TNBC. CTPS1 might be a promising prognosis biomarker and potential therapeutic target for patients with triple-negative breast cancer.

**Supplementary Information:**

The online version contains supplementary material available at 10.1186/s12967-021-03206-5.

## Introduction

Breast cancer (BC) is now the most frequently diagnosed malignancy and the leading cause of death from cancer in women worldwide [1]. Triple-negative breast cancer (TNBC) represents the most aggressive subtype of breast cancer characterized by the absence of estrogen receptor (ER), progesterone receptor (PR) and human epidermal growth factor 2 (HER2). Although TNBC only accounts for about 15–20% of all breast cancer patients, it has the worst outcome due to high invasiveness and unsatisfactory therapeutic efficacy [2, 3]. Up to now, the use of surgical resection and adjuvant/neoadjuvant chemotherapy is the main treatment strategy for triple-negative breast cancer [2, 4]. However, some patients might still fail to respond and lead to poor prognosis after conventional therapy [5]. Therefore, a deep-going investigation of the molecular mechanisms underlying TNBC oncogenesis and progression is urgently needed.

Cytidine nucleotide triphosphate synthase 1 (CTPS1) is a CTP synthase which catalyzes CTP biosynthesis from ATP, UTP and glutamine [6, 7]. This enzyme is a 591-amino-acid protein with an N-terminal synthetase domain and a C-terminal glutaminase domain. CTP synthase activity is an important step for DNA synthesis and cell cycle arrest [7, 8]. Increased CTPS activity was also reported in a variety of human cancers [9, 10]. CTPS1 has been demonstrated to be intensively involved in immune system by its capacity to sustain the proliferation of activated lymphocytes during immune response [11]. Up to now, few studies have investigated the role of CTPS1 on tumor development and progression. We previously conducted a proteomic study with surgical specimens form 24 triple-negative breast cancer patients. CTPS1 was one of the highly differently expressed proteins (DEPs) between TNBC tumor and corresponding para-tumor tissues [12]. However, the potential oncogenic function of CTPS1 on TNBC and the underlying mechanism for the association between CTPS1 and TNBC still remains unknown.

To address this issue, we firstly evaluated the expression of CTPS1 and determined its prognostic value by public databases and immunohistochemical (IHC) analysis. A series of in vitro and in vivo experiments were then performed to confirm the oncogenic role of CTPS1 in TNBC. In addition, we have revealed that CTPS1 could act as a novel transcriptional target of YBX1 and enrichment analysis of genes co-expressed with CTPS1 was also conducted to identify potential signaling pathways. Our findings provide novel insight of CTPS1 in the progression of TNBC and suggest a new theoretical basis for the prevention and treatment of patients with triple-negative breast cancer.

## Materials and methods

### Microarray data processing and clinical samples

The microarray datasets of breast cancer patients were extracted from the Gene Expression Omnibus (GEO) database (http://www.ncbi.nlm.nih.gov/geo/) and and the Cancer Genome Atlas (TCGA) database (https://portal.gdc.cancer.gov/). Three microarray gene expression datasets (GSE21653 [13], GSE31448 [14] and GSE45827 [15]) were obtained from the GEO database. A total of 210 TNBC patients with complete clinicopathological and follow-up information were retrospectively reviewed from Fujian Medical University Union Hospital between June 2013 and February 2018. All patients received total mastectomy or breast conserving surgery without neoadjuvant chemotherapy or radiotherapy and should receive at least six cycles of adjuvant chemotherapy after surgery. Disease-free survival (DFS) was defined as the time from the date of diagnosis to the date of clinical relapse (with histopathology confirmation or radiological evidence of tumor recurrence). Overall survival (OS) was defined as the time from the date of diagnosis until death from any cause. The follow-up deadline was March 1, 2021. This procedure was approved by the Research Ethics Committee of Fujian Medical University Union Hospital and informed consent was obtained from each participant.

### Immunohistochemistry (IHC) staining and evaluation

IHC staining analysis was performed on paraffin-embedded tissues to measure the protein expression of CTPS1 and YBX1 in all TNBC tissues and adjacent normal breast tissues according to the standard immunoperoxidase staining procedure. Briefly, slides were incubated with anti-CTPS1 (1:500; ab244492, Abcam) and anti-YBX1 (1:300, 20339-1-AP, Proteintech) according to the manufacturer’s instructions. The IHC staining scores of CTPS1 and YBX1 were evaluated by two independent pathologists blinded to the corresponding patients. The percentage of stained positive cells was scored from 1 to 4: 1, 0–25%; 2, 26–50%; 3, 51–75%; and 4, 75–100%. The staining intensity score was calculated from 0 to 3: 0, no staining; 1, weak staining; 2, moderate staining; and 3, strong staining. The percentage of positive tumor cells and the staining intensity were multiplied to produce a weighted score for each patient. A score of 8–12 was defined as high expression level and a score of 0–7 was defined as low expression.

### Cell culture and transfection

Human breast cancer cell lines (MDA-MB-231, HCC1937, BT-549, Hs578T, SKBR-3 and MCF-7) were purchased from the Cell Bank of Type Culture Collection of The Chinese Academy of Sciences. All cell lines were cultured in DMEM (HyClone) supplemented with 10% FBS (Gibco) and 1% penicillin and streptomycin solution at 37 °C under 5% CO2 conditions in a humidified incubator. Short hairpin RNA (shRNA) targeting CTPS1 were subcloned into GV115 and GV493 lentiviral shRNA vector (Genechem, Shanghai, China), respectively. For overexpressing YBX1, the construct was generated by subcloning PCR amplified full-length human YBX1 cDNA into the GV657 vector (Genechem, Shanghai, China). The constructed lentiviral vectors were packaged into the viruses in 293 T cells. Then, the harvested and concentrated viruses were added into cells and cultured for 72 h. The target sequences of the shRNA and negative control were as follows:

shCTPS1-1: 5’-ATCTTGTAGCGGATGATTC-3’.

shCTPS1-2: 5’-GAGGATTTGGTGTTCGAGGA-3’.

shCtrl: 5’-TTCTCCGAACGTGTCACGT-3’.

### RNA isolation and qRT-PCR analysis

Total RNA was extracted with TRIzol reagent (Invitrogen). Complementary DNA was synthesized by PrimeScript RT Master Mix (Takara) and qRT-PCR was subsequently performed on a model 7500 Real-Time PCR System (Applied Biosystems) with SYBR Green kit (Takara) following the manufacturer’s instructions. GAPDH gene was detected for normalization of data. Fold changes of gene expression were calculated by the 2 − ΔΔct method, three independent replicates of all biological samples were assessed. The primers used in qRT-PCR were listed in Additional file 2: Table S1.

### Western blotting

Total protein was extracted by RIPA lysis buffer (Beyotime) and the protein concentrations were determined with BCA Protein Assay Kits (Beyotime). A total of 20 µg of protein was loaded for electrophoretic separation on SDS/polyacrylamide gels and transferred onto polyvinylidene difluoride (PVDF) membrane. Membranes were blotted with the following antibodies: anti-CTPS1 (#98287, Cell Signaling Technology), anti-YBX1 (#9744, Cell Signaling Technology) and anti-GAPDH (ab181602, Abcam). Binding of the primary antibody was detected by incubating the membranes with a horseradish peroxidase-conjugated secondary antibody, followed by visualization with the ECL reagent (Thermo Fisher Scientific, Inc.).

### Cell proliferation assay

Cell growth and viability was detected by cell count kit-8 (CCK8) assay and colony formation assay. For CCK8 assay, cells were seeded into 96-well plates at the concentration of 2000 cells/well. Then, a 10 ul of Cell Counting Kit-8 (Dojindo, Japan) was added after 24, 48, 72, and 96 h of incubation, respectively. After 2 h, the absorbance was measured at 450 nm through a microplate reader. For colony formation assay, 1000 cells were seeded into a 6-well plate and continuously incubated for 14 days. The colonies were fixed with 4% paraformaldehyde for 30 min and stained with 0.1% of crystal violet solution (Sangon Bio, Inc.) for 15 min. Finally, the crystal violet stained colonies were counted to determine colony formation.

### Migration and invasion assays

Transwell assays were performed to detect cell migration and invasion. Cells were harvested, washed with PBS and suspended in DMEM without FBS at 1 × 10^5^ cells/ml. The upper chamber of the Transwell (Corning, Inc.) was filled with 100 µl of cell suspension, and the lower chamber was filled with 600 µl of DMEM with 30% FBS. Following incubation for 24 h at 37 °C, cells were fixed with 4% paraformaldehyde for 30 min and stained with 0.5% crystal violet for 5 min at room temperature. The chamber was then washed with PBS solution, and the cells on the surface of the chamber were wiped of with cotton swabs. The images of stained cells on the lower side were captured by a light microscope from 5 different randomly selected views under 200 × magnification. For invasion assays, transwell chambers precoated with Matrigel (BD Biosciences) for 2 h at 37 °C were utilized following a similar protocol as the cell migration assays.

### Apoptosis assay

An Annexin V-APC Apoptosis Detection Kit (eBioscience) was used for evaluation of cell apoptosis. Cells were seeded onto 6-well plates and grown to 70% confluence. After 72 h, the cells were harvested and washed following by 5 min of centrifugation at 1300 rpm. Cell pellets were subsequently resuspended and co-incubated with 10 ul of Annexin V-APC for 15 min in the dark at room temperature. Finally, the apoptosis rate was determined by a flow cytometer (Guava EasyCyte HT).

### Tumor Xenograft model

Female BALB/c nude mice (4 weeks), weighing approximately 20 g, were purchased from Vital River Laboratory Animal Technology Co. Ltd. (Beijing, China). The mice were kept in sterile cages with a controlled specific environment (22–25 °C, 40–60% relative humidity and a 12:12 h day/night light cycle). MDA-MB-231 cells (1 × 10^7^) stably transfected with shCTPS1 and negative control (shCtrl) were subcutaneously injected into the lower flank of the mice (n = 10 for each group). The size of tumor was measured twice a week for 4 weeks, and the tumor volume was calculated according to the formula: (length × width^2^)/2. After 4 weeks, all mice were sacrificed and the xenografts were dissected and weighed. The study protocol was approved by the Research Ethics Committee of Fujian Medical University Union Hospital. All procedures were performed in accordance with the Guide for the Care and Use of Laboratory Animals.

### Dual-luciferase reporter assay

MDA-MB-231 cells were seeded in 24-well plates at an 60% concentration and transfected with relevant plasmid and the luciferase vector. After 48 h, the activities of firefly and Renilla luciferases were measured by a Dual Luciferase Reporter Assay System (Promega) according to the manufacturer’s protocol. The luminescence intensities of firefly and Renilla luciferases were recorded by a microplate reader. For data analysis, the luciferase activity was measured relative to Renilla to standardize the background signal.

### Chromatin immunoprecipitation (ChIP) assay

The ChIP assay was conducted in MDA-MB-231 cells with the EZ ChIP™ Chromatin Immunoprecipitation Kit (Millipore, USA) according to the manufacturer’s instructions. The antibodies used in ChIP assay were anti-YBX1 (#9744, Cell Signaling Technology) and normal rabbit IgG (#2729, Cell Signaling Technology). The enriched DNA was analyzed by real-time PCR.

### Gene set enrichment analysis (GSEA)

The “sva” R package was applied to remove batch effects of the three GEO datasets (GSE21653, GSE31448 and GSE45827). Following standardization, the three independent datasets were combined. TNBC patients obtained from three GEO datasets and TCGA database were divided into high and low CTPS1 group according to the median CTPS1 expression level, respectively. GSEA was performed with the “clusterprofler” R package. Gene sets with NOM *p* < 0.05 and False Discovery Rate (FDR) q < 0.05 were considered to be significant.

### Weighted correlation network analysis (WGCNA)

WGCNA is a typical systems biology algorithm for identifying highly correlated genes and constructing gene co-expression networks. The “WGCNA” R package was used to indicate the highly correlated genes and co-expression networks of CTPS1 in TNBC patients. Firstly, network topology was calculated to choose the most appropriate soft threshold. An adjacency matrix was then built with the co-expression similarity matrix and a topological overlap matrix (TOM) was constructed. Finally, network modules were identified by dynamic hierarchical tree clustering. DAVID (http://david.abcc.ncifcrf.gov/) database was applied with gene KEGG pathway and GO functional enrichment analysis.

### Statistical analysis

Statistical analyses were performed by SPSS 20.0 software (IBM, United States) and GraphPad Prism 7.0 (GraphPad Software). Each experiment was repeated 3 times and presented as the means ± SD (standard deviation). A student’s *t*-test was performed to compare variables between two groups. The Chi-Square test was used to examine the clinicopathological characteristics between CTPS1 high and CTPS1 low expressing patients. Correlation between the expression levels of YBX1 and CTPS1 was analyzed by Spearman rank correlation coefficients. Survival curves were plotted by Kaplan–Meier method and analyzed by log-rank test. Cox proportional hazard regression model was applied for univariate and multivariate survival analysis. A two-sided *P* value of less than 0.05 was considered statistically significant.

## Results

### CTPS1 is highly expressed in TNBC tissue and predicts poor prognosis

**Fig. 1:**
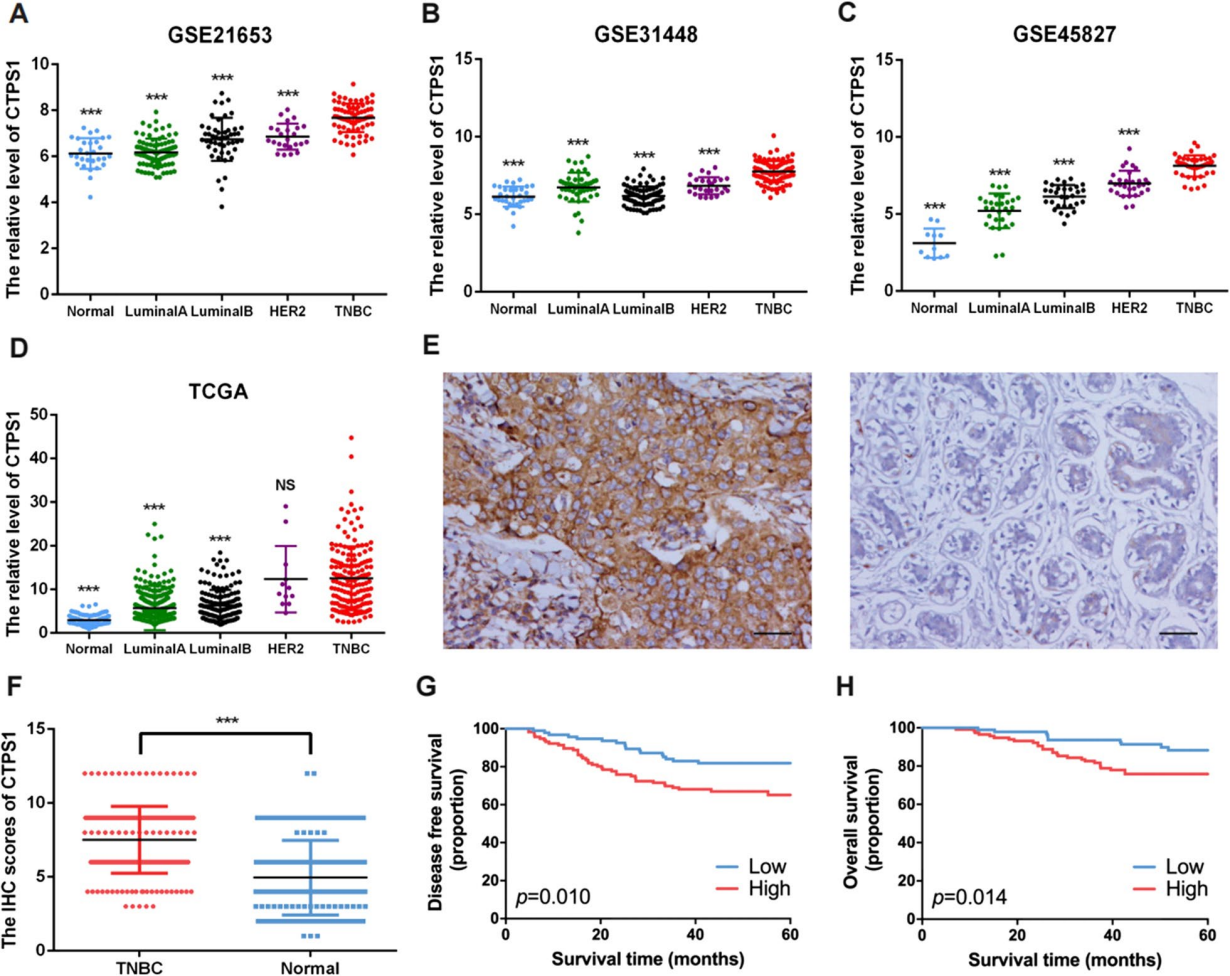
CTPS1 expression is elevated in TNBC and correlated with poor prognosis. **a**–**d** The mRNA expression of CTPS1 in different breast cancer subtypes and normal breast tissues was analyzed based on GEO (GSE21653, GSE31448, GSE45827) and TCGA databases. **e** Representative immunohistochemistry (IHC) images of CTPS1 in TNBC tumor and adjacent normal tissues (× 200). **f** The protein expression of CTPS1 in tumor and adjacent normal tissues from triple-negative breast cancer patients was detected by IHC. **g**, **h** Kaplan–Meier analysis of the disease-free survival (DFS) and overall survival (OS) with different CTPS1 expression in TNBC patients. Scale bar: 50um. *** *p* < 0.001

In this study, we firstly explored the CTPS1 expression levels in breast cancer tumor tissues and normal breast tissues in online databases. We performed data mining and analyzed CTPS1 mRNA profiles from the publicly available GEO datasets (GSE21653, GSE31448, GSE45827) and TCGA database. The results indicated that CTPS1 was highly expressed in TNBC tumor tissues than other breast cancer subtypes or normal breast tissues in all three GEO datasets (all *p* < 0.001, Fig. 1A–C). For TCGA database, the expression of CTPS1 was also significantly elevated in TNBC tumor tissues compared to LuminalA, LuminalB breast cancer subtype and normal breast tissues (*p* < 0.001, Fig. 1D). Next, immunohistochemical (IHC) staining assay was performed by 210 pairs of TNBC patient specimens and their corresponding adjacent normal tissues. We scored the CTPS1 expression level based on the CTPS1 staining intensity and percentage of positive tumor cells. The IHC score of CTPS1 expression was found to be significantly elevated in the TNBC tumor tissues than the noncancerous counterparts (Fig. 1E, F). Higher CTPS1 expression was also correlated with larger tumor size (*p* = 0.023), higher histological grade (*p* = 0.019) and lymphovascular invasion (*p* = 0.012) (Table 1). Furthermore, patients with high expression of CTPS1 exhibited a significantly poor disease-free survival (DFS) and overall survival (OS) compared to those with low expression in the Kaplan–Meier analysis (Fig. 1G, H). As for univariate and multivariate cox analyses, CTPS1 expression was determined to be an independent prognostic factor for both DFS and OS (HR = 1.90, 95%CI = 1.07–3.37, *p* = 0.029 and HR = 2.34, 95%CI = 1.13–4.84, *p* = 0.023, respectively, Table 2). Collectively, these results demonstrate that elevated CTPS1 expression is a risk factor for triple-negative breast cancer patients and could be considered as a potential prognostic biomarker.

**Table 1:** Associations of CTPS1 expression with clinicopathological characteristics for triple-negative breast cancer patients

Characteristics	All patients		Low CTPS1	High CTPS1	*p* value^a^
	n = 210		n = 94	n = 116	
	No	(%)	No	No	
Age at diagnosis(years)					0.114
≤ 50	99	47.1	50	49	
> 50	111	52.9	44	67	
Tumor size					0.023
≤ 2 cm	87	41.4	47	40	
> 2 cm	123	58.6	47	76	
Lymph node metastasis					0.154
No	125	59.5	61	64	
Yes	85	40.5	33	52	
Tumor Grade					0.019
I + II	80	38.1	44	36	
III	130	61.9	50	80	
Lymphovascular invasion					0.012
No	130	61.9	67	63	
Yes	80	38.1	27	53	

^a^The p value was calculated among all groups by the Chi-square test

**Table 2:** Univariate and multivariate cox proportional hazard model for disease free survival (DFS) and overall survival (OS) in TNBC patients

Variables	Univariate analysis				Multivariate analysis			
	DFS		OS		DFS		OS	
	HR (95% CI)	*P* ^a^	HR (95% CI)	*P* ^a^	HR (95% CI)	*P* ^a^	HR (95% CI)	*P* ^a^
Age (years)								
≤ 50	Reference		Reference					
> 50	0.65 (0.38–1.11)	0.114	0.95 (0.50–1.81)	0.870				
Tumor size								
≤ 2 cm	Reference		Reference		Reference			
> 2 cm	1.96 (1.10–3.50)	0.023	1.82 (0.90–3.67)	0.098	1.71 (0.95–3.07)	0.073		
Lymph nodes metastasis								
No	Reference		Reference		Reference		Reference	
Yes	1.82 (1.08–3.07)	0.026	2.00 (1.05–3.84)	0.036	1.65 (0.97–2.79)	0.065	1.85 (0.96–3.55)	0.066
Grade								
I + II	Reference		Reference					
III	1.50 (0.85–2.65)	0.164	1.75 (0.85–3.62)	0.130				
Lymphovascular invasion								
No	Reference		Reference					
Yes	1.66 (0.98–2.81)	0.057	1.47 (0.77–2.81)	0.240				
CTPS1 expression								
Low	Reference		Reference		Reference		Reference	
High	1.97 (1.10–3.55)	0.010	2.49 (1.20–5.15)	0.014	1.90 (1.07–3.37)	0.029	2.34 (1.13–4.84)	0.023

*HR* hazard ratio, *CI* confidence interval, *DFS* disease free survival, *OS* overall survival

^a^The *P* value was adjusted by the univariate Cox proportional hazard regression model

### CTPS1 knockdown suppresses proliferation, migration, invasion and induces apoptosis of TNBC cells in vitro

**Fig. 2:**
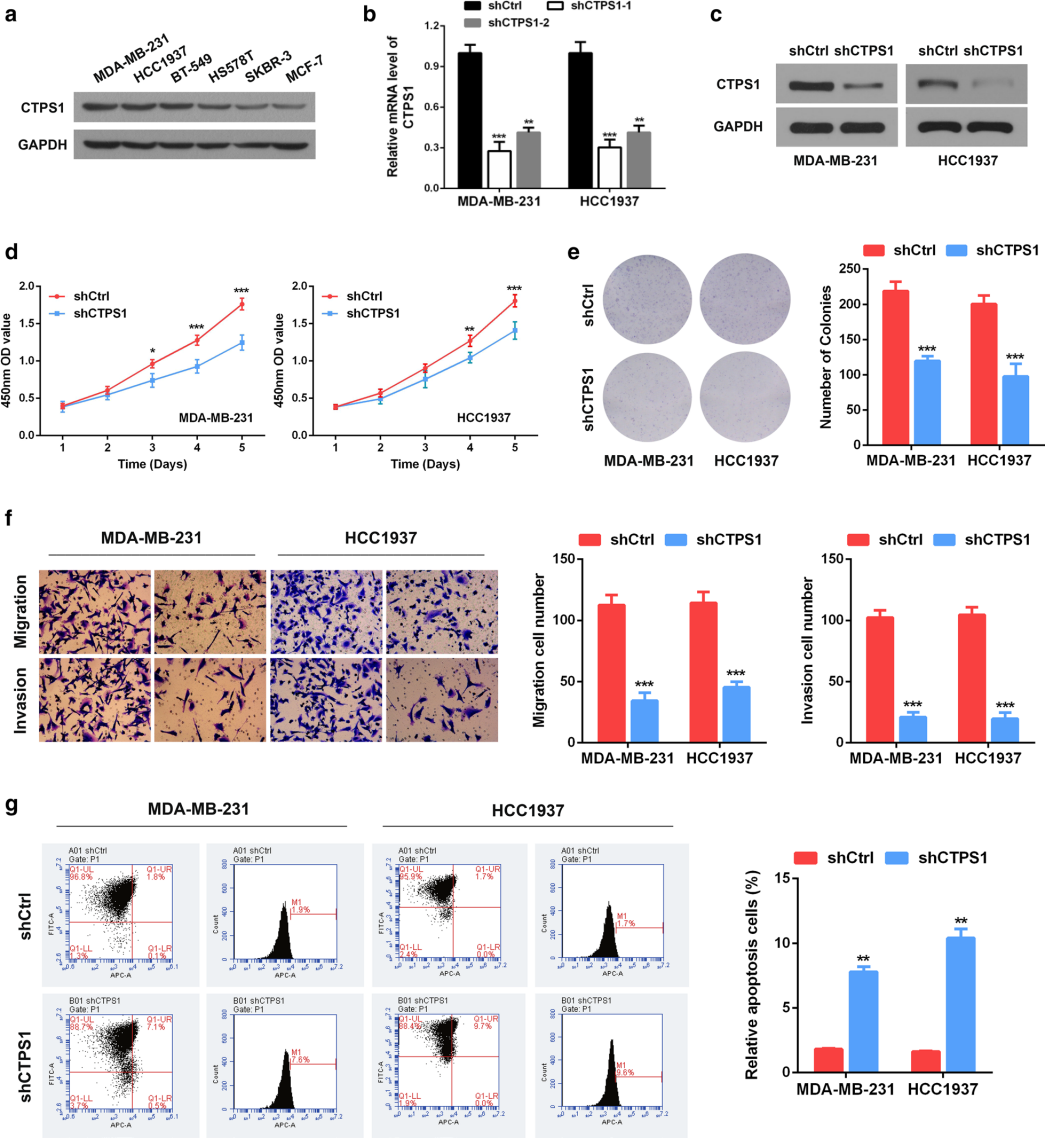
Knockdown of CTPS1 suppresses cell proliferation, migration, invasion and induces apoptosis of TNBC cells in vitro. **a** CTPS1 protein expression in TNBC cell lines was analyzed by western blot. **b** The knockdown efficiency of shCTPS1 was detected by qRT-PCR in MDA-MB-231 and HCC1937 cells. **c** The knockdown efficiency of shCTPS1 for following experiments was validated by western blot. **d**, **e** Cell proliferation was detected by CCK8 and colony formation assay following CTPS1 knockdown in TNBC cells. **f** Cell migration and invasion ability was determined by transwell assay following CTPS1 knockdown in TNBC cells. **g** Cell apoptosis of TNBC cells following CTPS1 knockdown was analyzed by flow cytometer. All data are presented as the mean ± SD of three independent experiments. * *p* < 0.05, ** *p* < 0.01, *** *p* < 0.001

To investigate the function role of CTPS1 in TNBC cells, we firstly measured CTPS1 levels in several breast cancer cell lines (Fig. 2A). CTPS1 was proved to be highly expressed in TNBC cell lines and we selected MDA-MB-231 and HCC1937 cell lines for further studies. Two shRNAs (shCTPS1-1 and shCTPS1-2) were designed and synthesized to knock down CTPS1 in both cell lines (Fig. 2B). The shRNA (shCTPS1-1) that exhibited higher interference efficiency was confirmed by western blotting and selected for the subsequent experiments (Fig. 2C). The expression of CTPS1 in MDA-MB-231 and HCC1937 cells was significantly suppressed compared with the shCtrl group. CCK-8 and colony formation assays indicated that CTPS1 silencing significantly impeded the viability and cloning ability of both MDA-MB-231 and HCC1937 cells (Fig. 2D, E). The capabilities of cell migration and invasion were also significantly decreased after CTPS1 knockdown by transwell assay (Fig. 2F). Moreover, we also observed dramatically increased apoptotic cells in shCTPS1 group compared with that in shCtrl group (Fig. 2G). In summary, these findings confirm that loss of CTPS1 could suppress proliferation, migration, invasion and induces apoptosis in TNBC cells.

### CTPS1 promotes TNBC development in vivo

**Fig. 3:**
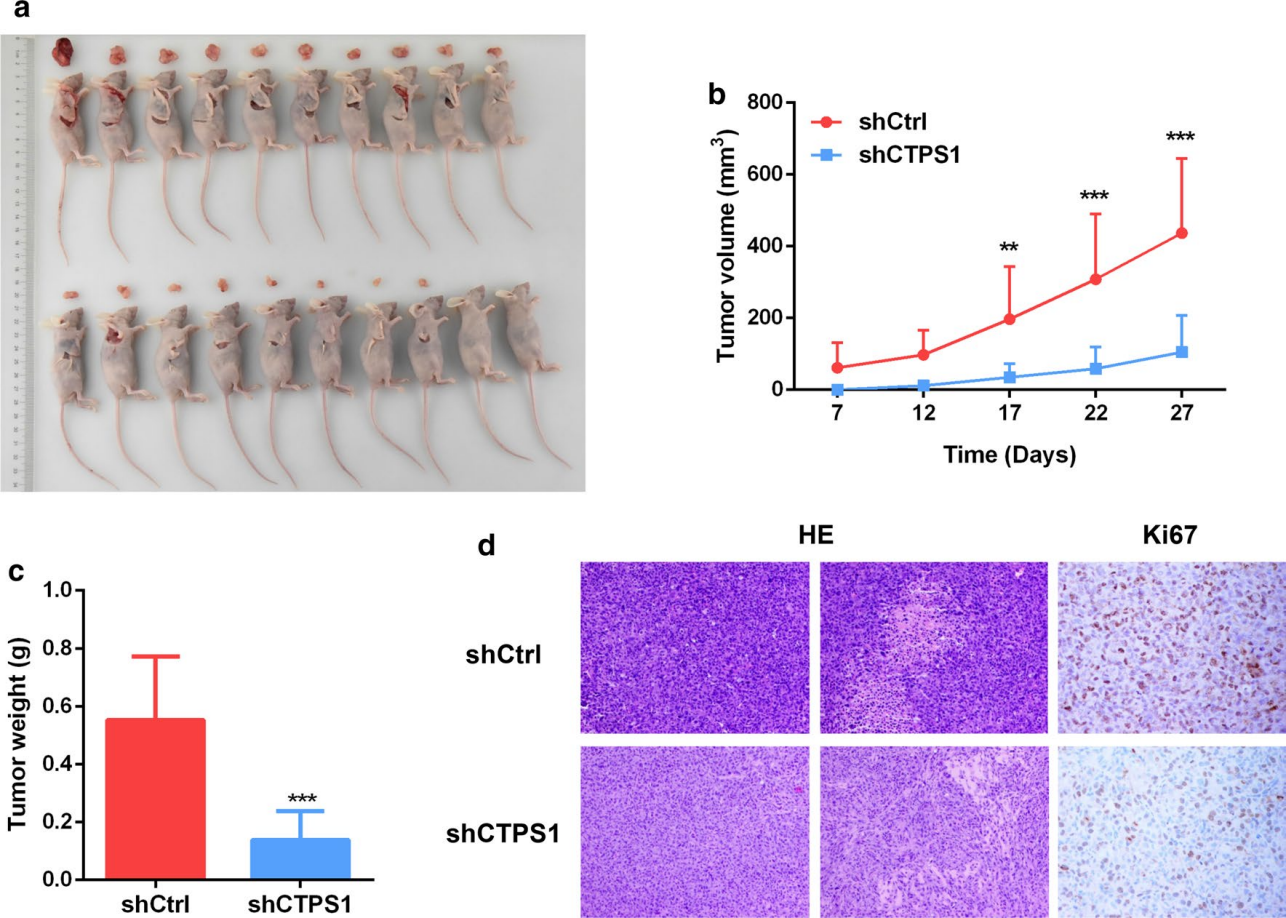
CTPS1 promotes TNBC tumor growth in vivo. **a** Images of xenograft tumors from groups of BALB/c-nude mice 4 weeks after the subcutaneous injection of stable CTPS1 knockdown MDA-MB-231 cells (shCTPS1) or control cells (shCtrl). **b** Tumor volume was calculated according to the formula: (length × width^2^)/2. **c** Tumor weight in nude mice of shCTPS1 and shCtrl group was assessed at day 28. **d** Hematoxylin–eosin (HE) staining and immunohistochemistry for proliferation marker Ki67 in xenograft tumors. ** *p* < 0.01, *** *p* < 0.001

To validate the function of CTPS1 on triple-negative breast cancer, TNBC xenograft tumor model in nude mice was established to evaluate the effect of CTPS1 knockdown in vivo. The nude mice were subcutaneously injected with MDA-MB-231 cells with stable CTPS1 knockdown (shCTPS1) or with negative control (shCtrl), respectively. The size of tumor was measured twice a week after cell injection and the mice were sacrificed after 4 weeks. As shown in Fig. 3A, B, the tumor volume was drastically suppressed by the inhibition of CTPS1. The tumor weight was also found to be remarkably decreased in shCTPS1 group after CTPS1 knockdown (Fig. 3C). In addition, both hematoxylin–eosin (HE) staining and proliferation marker Ki67 showed decreased cell mitosis and lower percentage of proliferative cells in the shCTPS1 group (Fig. 3D). All these data provide evidence that CTPS1 could act as a tumor activator for the growth of TNBC in vivo.

### YBX1 activates CTPS1 transcription in TNBC cells

**Fig. 4:**
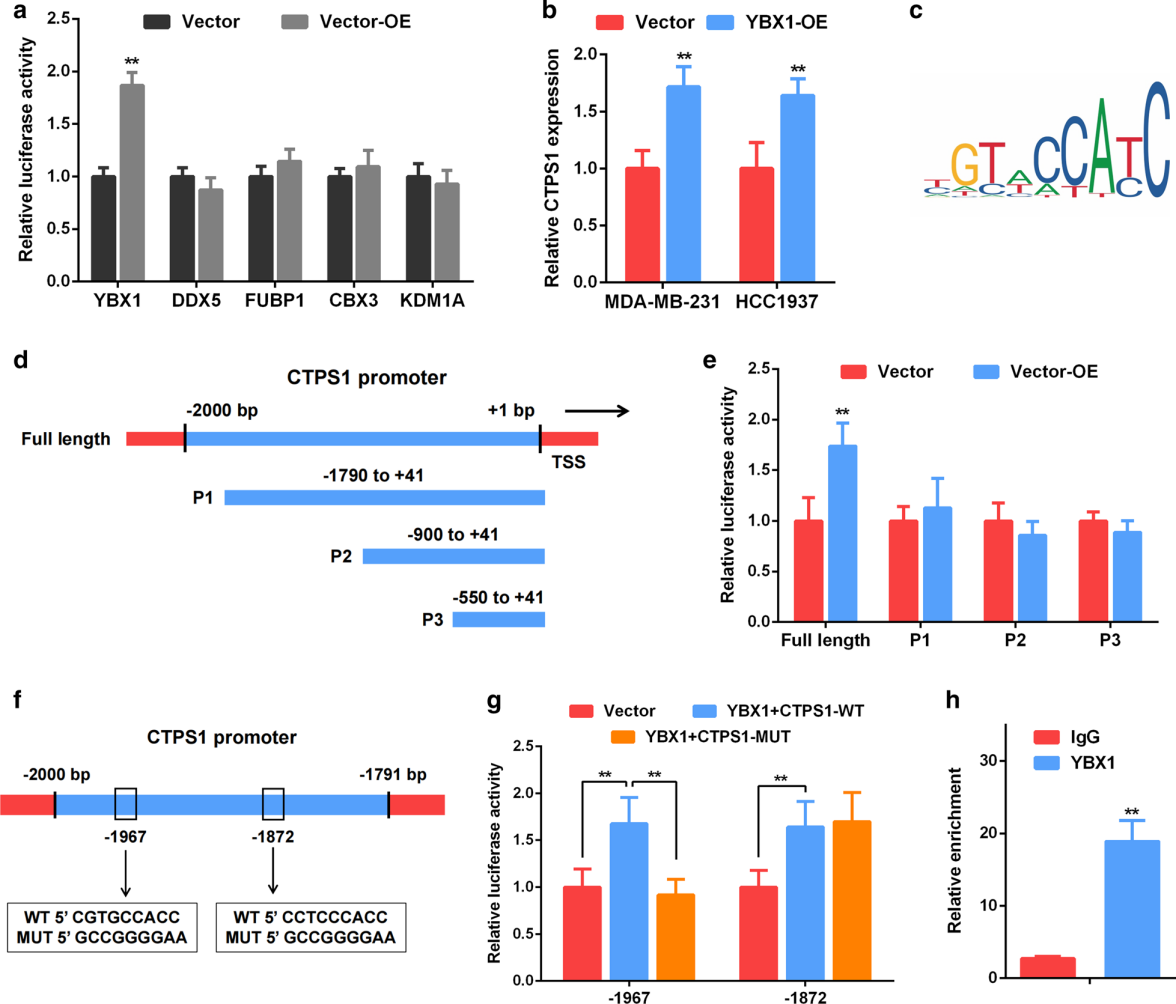
YBX1 directly binds to the CTPS1 promoter to regulate CTPS1 expression in TNBC cells. **a** Dual-luciferase reporter assay confirmed the interaction of YBX1 and CTPS1 promoter in MDA-MB-231 cells. **b** CTPS1 mRNA expression level was identified by qRT-PCR after overexpression of YBX1. **c** The binding site of YBX1 and CTPS1 was predicted by JASPAR database. **d** Diagrammatic drawing of CTPS1 promoter fragments. **e** Luciferase activity of different CTPS1 promoter fragment was detected after YBX1 overexpression. **f**, **g** Luciferase activity of CTPS1 promoter (WT or MUT) was detected after YBX1 overexpression in MDA-MB-231 cells. **h** ChIP assay verified that YBX1 could bind to the CTPS1 promoter in MDA-MB-231 cells. All data are presented as the mean ± SD of three independent experiments. ** *p* < 0.01

To further explore the molecular mechanism of CTPS1 in triple-negative breast cancer, we used JASPAR database (http://jaspar.genereg.net/) and PROMO software (http://alggen.lsi.upc.es/cgi-bin/promo_v3/promo/promoinit.cgi?dirDB=TF_8.3) to predict potential transcription factors of CTPS1 [16–18]. Five candidates including YBX1, DDX5, FUBP1, CBX3 and KDM1A were obtained for further analysis. By utilizing a luciferase reporter construct, only the relative luciferase activity of CTPS1 (within 2000 bp promoter region) was significantly increased after transfection with YBX1 overexpression plasmid (Fig. 4A). In addition, overexpression of YBX1 also significantly increased the mRNA expression of CTPS1 in MDA-MB-231 and HCC1937 cells (Fig. 4B). Therefore, YBX1 was identified as the most possible transcription factor that interacts with CTPS1. Subsequently, the specific YBX1 binding sites on the CTPS1 promoter was also predicted (Fig. 4C) and listed in Additional file 3: Table S2. According to the predicted binding sites, we designed three CTPS1 promoter fragments and named from P1 to P3 (− 1740 to + 41 bp, − 900 to + 41 bp, −550 to + 41 bp, Fig. 4D). The relative luciferase activity of each fragment had no significant changes after co-transfection (Fig. 4E). Thus, two predicted binding sites (−1967, CGTGCCACC and −1872, CCTCCCACC) were selected and the enhanced luciferase activity was reversed by transfection of “CGTGCCACC” mutated CTPS1 sequence (Fig. 4F, G). Moreover, ChIP assay was performed and indicated that YBX1 was significantly enriched in the CTPS1 promoter in TNBC cells (Fig. 4H). Taken together, YBX1 could directly activate CTPS1 transcription by binding to its promoter region in triple-negative breast cancer.

### YBX1/CTPS1 axis is involved in the progression of TNBC

**Fig. 5:**
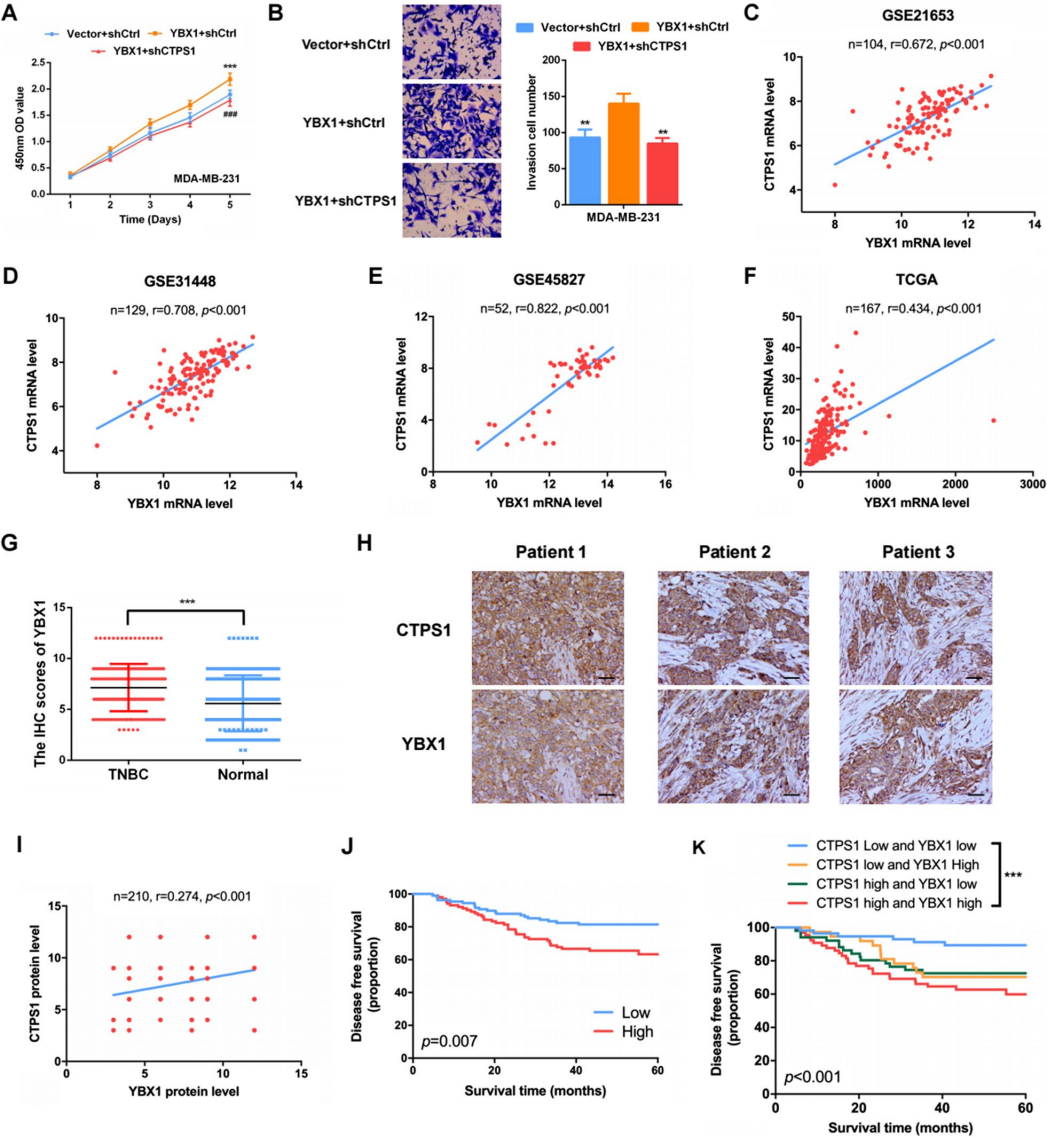
YBX1/CTPS1 axis is involved in the progression of TNBC. **a**, **b** Cell proliferation and invasion ability was assessed by CCK-8 and transwell assay after overexpression of YBX1 and downregulation of CTPS1. All data are presented as the mean ± SD of three independent experiments. **c-f** The correlation between YBX1 and CTPS1 mRNA expression was evaluated by Spearman’s correlation analysis based on GEO (GSE21653, GSE31448, GSE45827) and TCGA databases. **g** The protein expression of YBX1 in tumor and adjacent normal tissues from triple-negative breast cancer patients was detected by IHC analysis. **h** Representative staining images of CTPS1 and YBX1 in TNBC tumor tissues (× 200). **i** The correlation between YBX1 and CTPS1 protein expression was evaluated by Spearman’s correlation analysis based on IHC analysis. **j** Kaplan–Meier analysis of the disease-free survival with different YBX1 expression in TNBC patients. **k** Kaplan–Meier analysis of the disease-free survival with different YBX1 and CTPS1 expression in TNBC patients. Scale bar: 50um. ** *p* < 0.01, *** *p* < 0.001, ^###^*p* < 0.001 (YBX1 + shCtrl vs YBX + CTPS1)

YBX1 (Y-box binding protein-1) is an important transcription factor for tumor progression. Previous study has indicated that silencing of YBX1 could significantly reduce the invasive potential of TNBC [19]. To demonstrate the effects of YBX1/CTPS1 axis on triple-negative breast cancer, YBX1 vector and CTPS1 shRNA was co-transfected into MDA-MB-231 cells. CCK8 and transwell assays confirmed that overexpression of YBX1 could promote cell proliferation and invasion of TNBC cells (Fig. 5A, B). Rescue experiments identified that the enhanced cell proliferation and invasion ability induced by YBX1 overexpression could be reversed by CTPS1 knockdown (Fig. 5A, B). Next, we assessed the relationship between CTPS1 and YBX1 through Spearman’s correlation analysis by GEO datasets (GSE21653, GSE31448, GSE45827), TCGA database and 210 pairs of TNBC tissues. As shown in Fig. 5C–F, the mRNA level of YBX1 was positively correlated with CTPS1 in all four databases (*p* < 0.001). Similarly, IHC staining analysis revealed that YBX1 was elevated in TNBC tissues compared with adjacent non-cancer tissues (Fig. 5G, Additional file 1: Fig. S1A). The increased protein level of YBX1 also showed a significant correlation with that of CTPS1 (r = 0.274, *p* < 0.001, Fig. 5H, I). Survival curve analysis indicated that TNBC patients with high YBX1 expression had a relatively worse prognosis compared with those with low YBX1 expression (Fig. 5J, Additional file 1: Fig. S1B). Moreover, TNBC patients with high expression levels of both CTPS1 and YBX1 had a significantly poorer prognosis (Fig. 5K, Additional file 1: Fig. S1C). The above data suggest that YBX1 could affect TNBC prognosis along with CTPS1 and YBX1/CTPS1 axis is involved in the progression of triple-negative breast cancer.

### Gene set enrichment analysis of interrelated pathways and identification of key module by weighted correlation network analysis

**Fig. 6:**
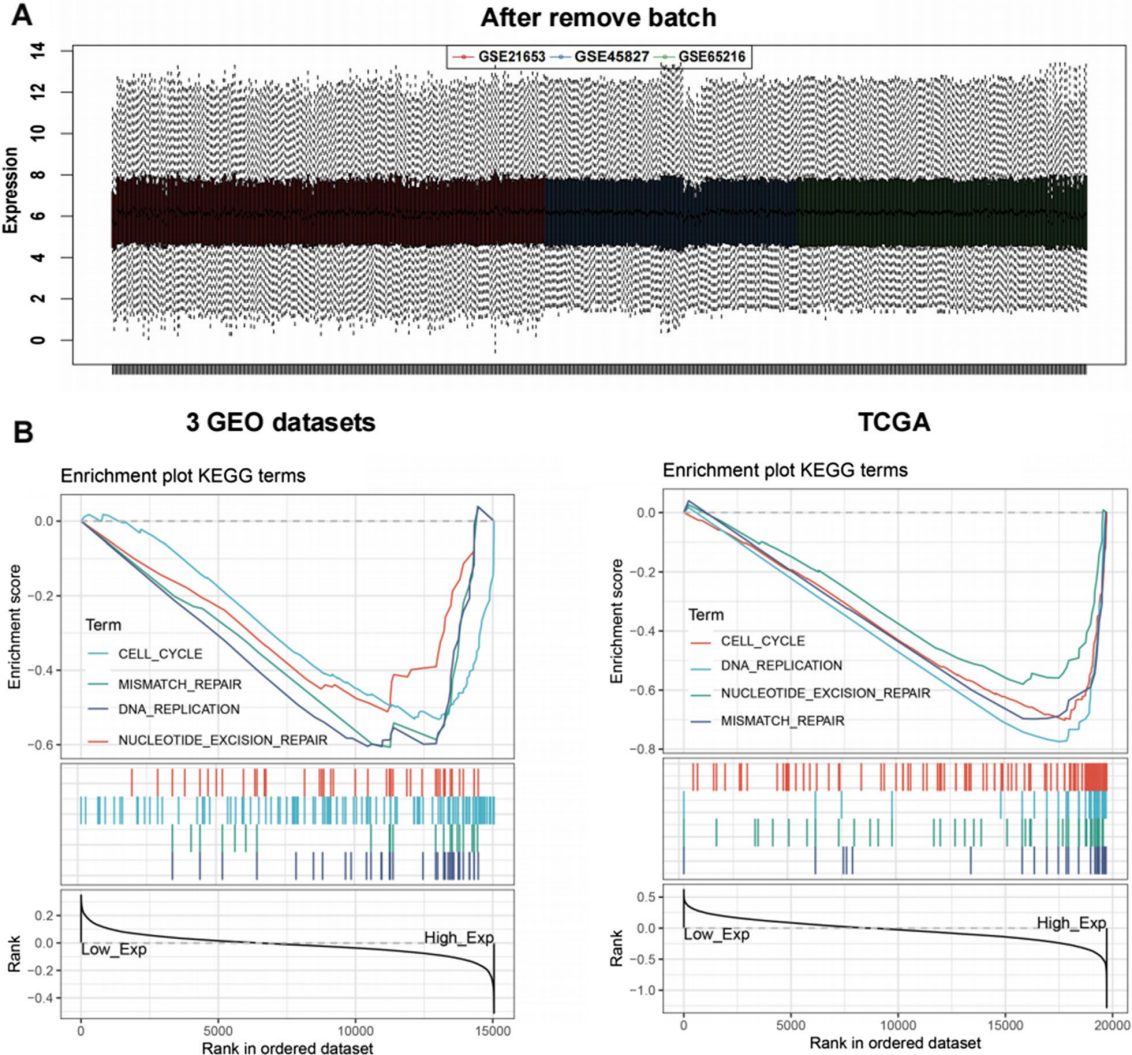
GSEA revealed interrelated pathways for CTPS1. **a** The distribution of the 3 selected GEO datasets after adjusting for batch effects. **b** KEGG terms of GSEA analysis for CTPS1 positively enriched functions by enrichment scores (ES) in 3 GEO datasets and TCGA database

To further explore the potential biological functions of CTPS1 in TNBC, batch effects were removed from three GEO datasets (Fig. 6A). After standardization, the three independent databases were combined. According to the median expression level of CTPS1, we divided TNBC patients into the high CTPS1 and low CTPS1 group in three GEO datasets and TCGA database, respectively. KEGG terms of GSEA indicated that higher CTPS1 expression was closely correlated with cell cycle, DNA replication, mismatch repair and necleotide excision repair (Fig. 6B).

**Fig. 7:**
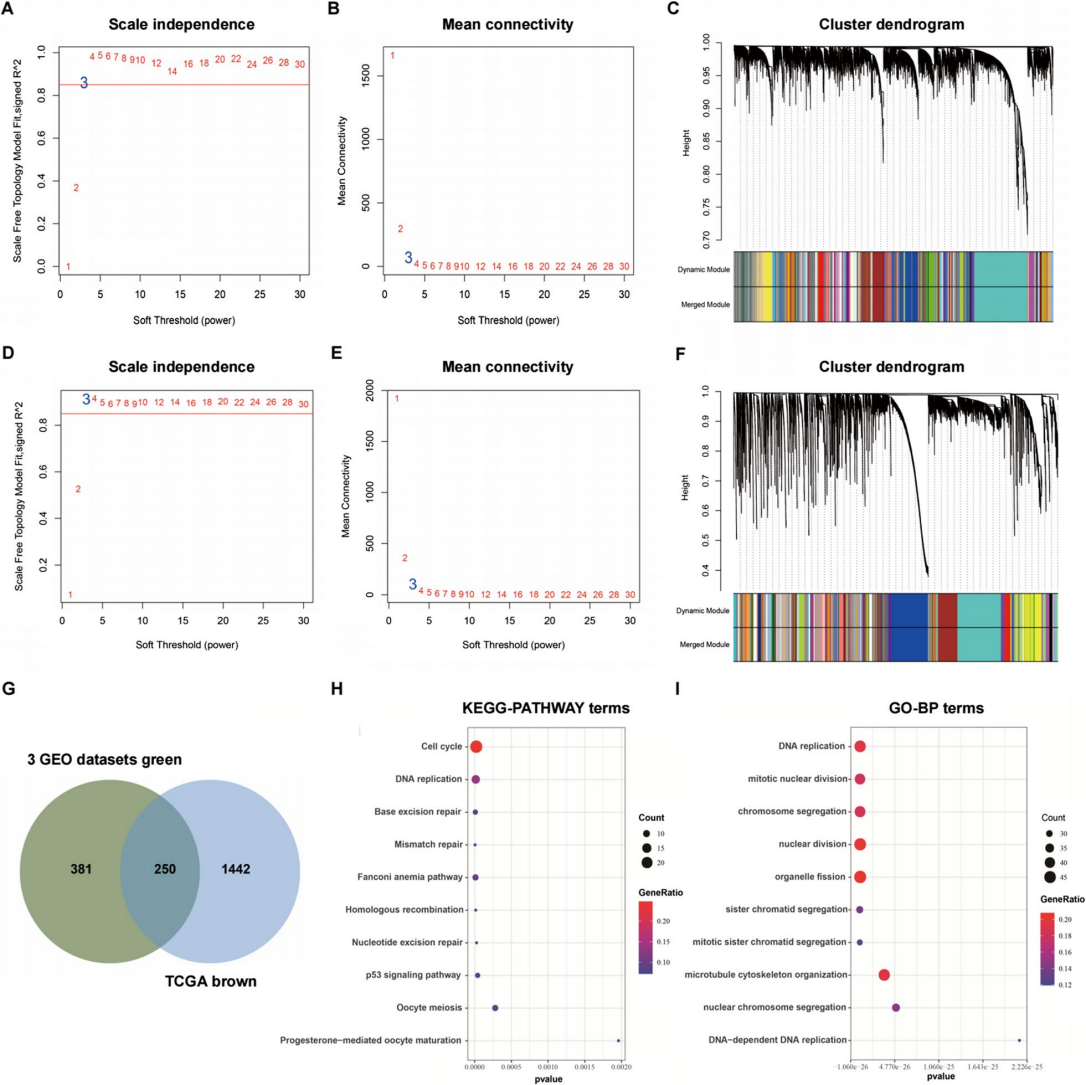
Identification of co-expression module genes associated with CTPS1 with the WGCNA. **a**, **d** Relationship between scale-free topology model fit and soft-thresholds (powers) in 3 GEO datasets and TCGA database. **b**, **e** Relationship between the mean connectivity and various soft-thresholds in 3 GEO datasets and TCGA database. **c**, **f** Dendrogram of modules identified by WGCNA in 3 GEO datasets and TCGA database. **g** The intersection of green module for 3 GEO datasets and brown module for TCGA database contained 250 co-expression module genes of CTPS1. **h**, **i** KEGG pathway and GO-BP terms for 250 co-expression module genes of CTPS1

For WGCNA, we firstly calculated the network topology and the power value 3 was the lowest power for the scale-free topology in 3 GEO datasets (Fig. 7A, B) and TCGA database (Fig. 7D, E). Afterwards, by selecting 3 as the soft threshold, a topological overlap matrix (TOM) was calculated (Fig. 7C, F). Finally, 100 meaningful modules for 3 GEO datasets and 76 meaningful modules for TCGA database were yielded by dynamic hierarchical tree clustering (Additional file 4: Table S3, Additional file 5: Table S4). The genes co-expressed with CTPS1 belonged to the green module containing 631 genes in 3 GEO datasets and brown module containing 1692 genes in TCGA database. The intersection of green and brown module consisted of 250 genes (Fig. 7G). KEGG analysis showed that the gene modules were enriched in cell cycle, DNA replication, base excision repair and mismatch repair (Fig. 7H). GO analysis results indicated that the terms of biological processes (BP) were DNA replication, mitotic nuclear division, chromosome segregation and nuclear division (Fig. 7I).

## Discussion

Triple-negative breast cancer (TNBC) is one specific subtype of breast cancer with high invasiveness and poor outcome. Although some progress has been made in TNBC, there are still no effective therapeutic targets to date. The most common treatment strategy for TNBC now is a combination of surgery, chemotherapy and radiotherapy [20–22]. Hence, identification of new regulatory molecules and promising therapeutic targets is of great importance in the treatment of patients with triple-negative breast cancer.

Cytidine nucleotide triphosphate synthase 1 (CTPS1) is a CTP synthase which catalyzes CTP biosynthesis from ATP, UTP and glutamine [6, 7]. Previous study has demonstrated that CTPS1 could represent a therapeutic target of immunosuppressive drugs against lymphocyte activation [11]. Few studies to date have evaluated its role in tumor development and progression. Our previous proteomic analysis has uncovered that CTPS1 is significantly highly expressed in TNBC tumor compared with corresponding para-tumor tissues [12]. However, the potential oncogenic function and precise mechanisms in TNBC warrant further study. In this study, CTPS1 expression was found to be upregulated in sample datasets procured from online GEO databases, TCGA database as well as in TNBC tissues by immunohistochemical (IHC) staining method. Higher CTPS1 expression was closely related with worse clinicopathologic features such as larger tumor size, higher histological grade and lymphovascular invasion. Moreover, lower expression of CTPS1 was associated with a better prognosis of patients with triple-negative breast cancer. These results collectively indicate that CTPS1 could act as a promising biomarker for the diagnosis and prognosis of TNBC patients. To further investigate the function role of CTPS1 in TNBC, we also performed a series of in vitro experiments and found that CTPS1 inhibition could dramatically inhibit the cell proliferation, migration, invasion and promoted cell apoptosis ability of TNBC cells. In addition, in vivo studies with mouse models revealed that CTPS1 knockdown remarkably reduced the tumor volume and weight. These data confirm the basic foundation role for CTPS1 as a new target in tumorigenesis and metastasis of TNBC.

To verify the potential transcriptional regulation that affected the overexpression of CTPS1 in TNBC, we used PROMO software and JASPAR database to predict the transcriptional factors that could regulate the expression of CTPS1. Five potential transcription factors including YBX1, DDX5, FUBP1, CBX3 and KDM1A were served as candidates. By utilizing the dual-luciferase reporter system, only YBX1 showed a higher relative luciferase activity of the CTPS1 promoter. ChIP-qPCR assay clearly revealed that YBX1 could directly regulate CTPS1 transcription by binding to its promoter. Moreover, YBX1-enhanced promoter activities were markedly abolished when the binding site was mutated, further demonstrating that YBX1-induced CTPS1 promoter activity was YBX1-dependent. Y-box binding protein 1 (YBX1), also known as YB-1, is a multifunctional protein that regulates transcription by binding to the Y-box (an inverted CCAAT box) at the promoter or enhancer of target genes [23]. As a RNA-binding protein, YBX1 plays essential roles in multiple aspects of RNA dynamic, including pre-mRNA splicing, mRNA packaging and translational regulation [23–26]. Numerous studies have suggested YBX1 could act as an oncoprotein in a variety of human cancers, including pancreatic cancer [27], colorectal cancer [28, 29], lung cancer [30, 31] and nasopharyngeal cancer [32, 33]. For breast cancer, YBX1 has been regarded as potential biomarker with poor outcome and silencing YBX1 could inhibit invasive potential through binding its downstream target, such as CORO1C and MMP1 [19, 34–36]. In our study, we also identified that overexpression of YBX1 could promote cell proliferation and invasion of TNBC cells, while rescue experiments indicated that the enhanced cell proliferation and invasion ability induced by YBX1 overexpression could be reversed by CTPS1 knockdown. Next, we further evaluated the associations between YBX1 and CTPS1 in TNBC by public databases and surgical specimens with IHC staining method. The mRNA and protein level of YBX1 was found to be highly correlated with CTPS1. Besides, patients with higher expression levels of both CTPS1 and YBX1 had a worse disease-free survival and overall survival compared with other patients. Altogether, these results firstly confirm the abiity of YBX1 to bind to the CTPS1 promoter and promote CTPS1 expression by increasing its transcriptional activity. The association between YBX1 and CTPS1 offers a novel approach by which TNBC could be targeted.

To further elucidate the functional role of CTPS1 in triple-negative breast cancer, we also conducted GSEA and WGCNA analysis with TNBC samples from GEO and TCGA databases to screen the gene and pathway sets related with CTPS1 expression. GSEA demonstrated that higher CTPS1 expression was closely correlated with cell cycle, DNA replication, mismatch repair and necleotide excision repair. The most significant module related with CTPS1 expression was identified by WGCNA, consisting of 631 genes in 3GEO databases and 1692 genes in TCGA database. After intersection, 250 genes were selected as hub module genes. Following the results of KEGG and GO analysis, the gene modules were principally enriched in cell cycle, DNA replication and nuclear division. These findings cooperate to indicate that CTPS1 might play a noticeable role in cell cycle regulation of triple-negative breast cancer.

## Conclusions

In summary, our study is the first exploration of the cytidine nucleotide triphosphate synthase 1 (CTPS1) in tumorigenesis. We observed that CTPS1 was overexpressed in TNBC tumor tissues and associated with poor prognosis. CTPS1 could promote carcinogenesis and metastasis of TNBC through transcriptional activation by YBX1. YBX1/CTPS1 is an important axis in the progression of triple-negative breast cancer. Taken together, CTPS1 might be a promising prognosis biomarker and potential therapeutic target for TNBC treatment.

## Supplementary Information

**Additional file 1: Fig. S1**. YBX1 expression is elevated in TNBC and correlated with poor prognosis. a Representative immunohistochemistry (IHC) images of YBX1 in TNBC tumor and adjacent normal tissues (×200). b Kaplan-Meier analysis of the overall survival with different YBX1 expression in TNBC patients. c Kaplan-Meier analysis of the overall survival with different YBX1 and CTPS1 expression in TNBC patients. Scale bar: 50um.

**Additional file 2: Table S1**. A list of primers used in this study.

**Additional file 3: Table S2**. The prediction binding domain between YBX1 and CTPS1.

**Additional file 4: Table S3**. Meaningful modules for 3 GEO datasets.

**Additional file 5: Table S4**. Meaningful modules for TCGA database.

## Data Availability

The datasets during and/or analysed during the current study are available from the corresponding author on reasonable request.
