# Supplementary figures and images for: CTPS1 promotes malignant progression of triple-negative breast cancer with transcriptional activation by YBX1
Source: J Transl Med. 2022 Jan 6;20:17. doi: 10.1186/s12967-021-03206-5 (PMC8734240; doi:10.1186/s12967-021-03206-5)

**A**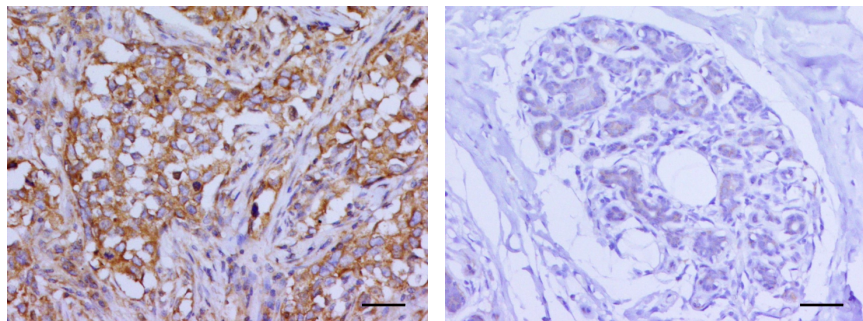**B**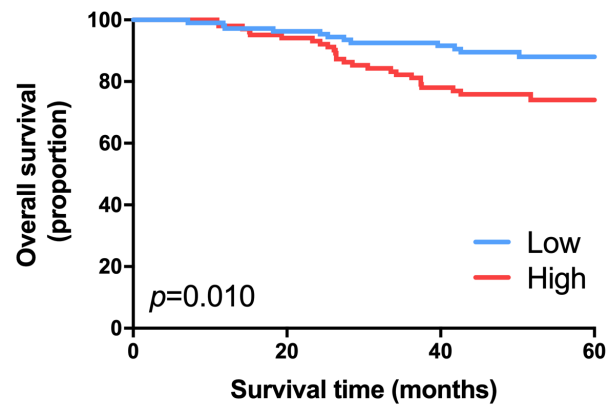**C**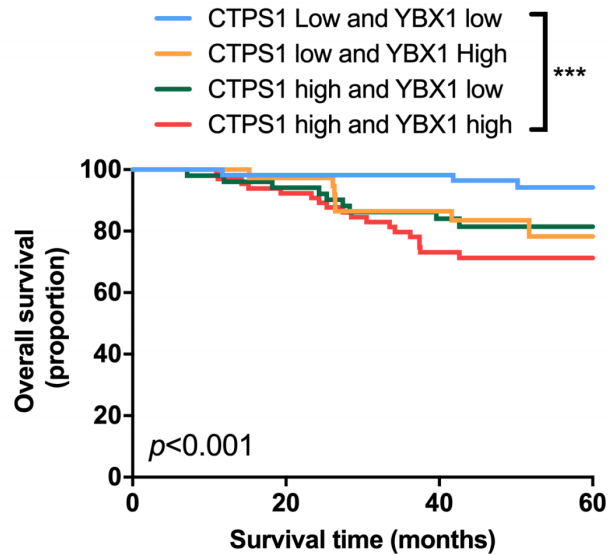

Supplement: Supplementary file 1 — Additional file 1: Fig. S1. YBX1 expression is elevated in TNBC and correlated with poor prognosis. a Representative immunohistochemistry (IHC) images of YBX1 in TNBC tumor and adjacent normal tissues (×200). b Kaplan-Meier analysis of the overall survival with different YBX1 expression in TNBC patients. c Kaplan-Meier analysis of the overall survival with different YBX1 and CTPS1 expression in TNBC patients. Scale bar: 50um. [file 12967_2021_3206_MOESM1_ESM.pdf]
